# Poisoning by Arsenic—Magnesia as an Antidote

**Published:** 1848-09

**Authors:** 


*Poisoning by Arsenic—Magnesia as an Antidote.*—M. Cadet-Gassicourt has related two cases of arsenical poisoning, in which hydrated magnesia was successfully administered. Both cases occurred in the practice of M. Chammartin. (*Journal de Chime Medicale, Fevrier et Mars,* 1848.)

The subject of the first case was a lady in Paris, who, on the 27th of October, 1847, took a considerable dose of arsenic, in the form of a powder sprinkled on bread and butter. Three or four hours after this she took a cup of coffee; this brought on immediate vomiting, which recurred at intervals. Between six and seven o'clock the same evening M. Chammertin was called in, and found the patient suffering from all the symptoms of arsenical poisoning. He prescribed hydrated oxide of magnesia, of which 300 grammes (between ℥ix and ℥x), were given in the course of two hours. It was followed by liquid evacuations, and the patient recovered.

The subject of the second case was a man æt. 23, of dissipated habits. Three hours after an unusually full supper he took a large dose of powdered arsenic, followed by copious draughts of water. He passed the night in great agony in the bowels and chest, but had no nausea, vomiting, or diarrhœa. At 11 A. M. the next day, M. Chammartin was sent for, and found the man in a state of great collapse, with his face pale, and his features haggard and pinched; he was agitated and spoke with a feeble voice; his respiration was difficult, and he complained of a tearing sensation along the gullet and at the epigastrium, and of thirst and dryness of the fauces. His tongue was moist, but red at the edges and point, his deglutition was easy, and there was no diarrhœa, though he suffered from colics and cramps in all his limbs. The hydrated oxide of Magnesia was then given, warmth was applied to the surface, and he was afterwards bled. He was then removed to the Hotel Dieu, where all his symptoms improved. He finally recovered. The quantity of magnesia given was about 500 grammes (about ℥xvij), but while in the Hotel Dieu some hydrated sesquioxide of iron was administered.

The same journals contain an account of the dispute between MM. Caventon and Bussy, as to the comparative efficacy of the hydrated magnesia, and the hydrated sesquioxide of iron as antidotes to arsenic. The former advocates the superiority of the sesquioxide over the magnesia, while M. Bussy is inclined to the opposite opinion. M. Caventon says that the salt formed by the iron with the arsenic is less likely to be decomposed by the muriate of ammonia which naturally exists in the stomach and intestines, and states that this salt readily decomposes the arsenite of magnesia, so that when the last is the antidote used, the arsenic is more liable to be reduced to a soluble state. But M. Bussy remarks that such a result is obviated by using an excess of magnesia, which again, according to M. Caventon, is apt to occasion an extrication of free ammonia, which, from its irritating properties, cannot but concur in complicating the case.

The same subject has also been investigated by M. Riegel, whose results are briefly these:—(*See Jahrbuch fur Prakt. Pharm. xiii., and Chemical Gazette, Aug.* 1, 1847.)

The author has been enabled to detect traces of arsenic in a filtered solution, upon the application of the sesquioxide of iron, by the sulphuretted hydrogen test, wherever the quantity of the oxide was less than seven parts, but the liquid was perfectly free from all traces of the poison wherever the antidote was added in the proportion of more than ten parts. With arsenic acid at least twelve parts were required to precipitate the acid completely. With respect to the hydrate of magnesia, he found that to precipitate entirely one part of arsenious acid, at least eighteen parts of the antidote are required, and he recommends that, in preparing the magnesia, one hundred parts of the sulphate should be precipitated by fifty parts of caustic potash, and the precipitate washed and preserved in bottles under water. Moreover, the author found that the compound formed by magnesia with arsenious acid was quite insoluble in cold and boiling water. When the arsenic is in combination with alkalies, this antidote does not completely remove it from its solution, but by mixing some undecomposed magnesian salt with the hydrate of magnesia, all traces of the poison were removed. He accordingly recommends a mixture of hydrate of magnesia and sulphate in water, in equal parts, as the most advantageous form, especially when it is uncertain whether the poisoning has resulted from free arsenious acid, or some alkaline arsenite.

In preparing the magnesia for the purpose of an antidote, it is necessary according to M. Bussy, to avoid calcining too much, as highly calcined magnesia is useless.—*Bouchardat, in Nouvelle Encyclographie des Sciences Med. Fevrier*, 1847.

